# A review of autophagy in the pancreas: normal physiology and pathophysiology

**DOI:** 10.1080/27694127.2026.2665907

**Published:** 2026-05-12

**Authors:** Miles Piper, Conan Kinsey

**Affiliations:** a Department of Oncological Sciences, University of Utah, Salt Lake City, UT, USA; b Department of Internal Medicine, University of Utah, Salt Lake City, UT, USA

**Keywords:** Autophagy, pancreas, pancreatitis, diabetes, pancreatic cancer

## Abstract

Autophagy is a complex cellular process of cellular degradation that is essential for healthy pancreatic function and, when perturbed, can result in pathological states such as pancreatitis, diabetes, and cancer. Extremes in both activation and inhibition can lead to inflammation, cell damage, and organ dysfunction. Pharmacologic targeting of autophagy to restore homeostasis may provide a therapeutic benefit to various pancreatic pathological processes. In this review, we discuss the current understanding of how autophagy maintains normal pancreatic function and is perturbed in various pancreatic disease states, including opportunities for potential therapeutic intervention.

## Introduction

**Figure 1.**
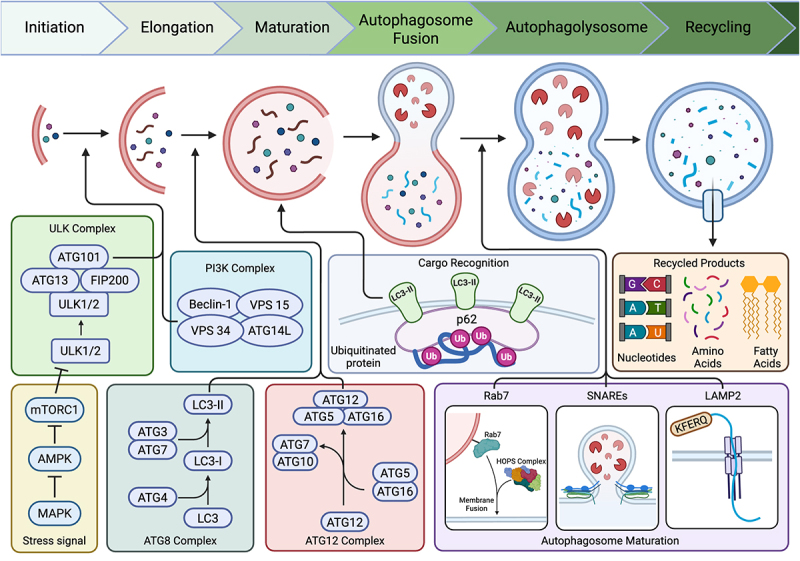
Canonical macroautophagy pathway and core regulatory complexes. Overview of stages of autophagy initiation, elongation, including initiation, phagophore elongation, autophagosome maturation, autophagosome – lysosome fusion, and degradation/recycling. Autophagy initiation is driven by stress-sensing pathways, including mTORC1 inhibition, AMPK activation, and/or MAPK inhibition, culminating in the activation of the ULK1/2 complex. Autophagophore nucleation requires the class iii PI3K complex (Beclin-1, VPS34, VPS15, ATG14L), while elongation is mediated by the ATG8/LC3 conjugation system and ATG12–ATG5–ATG16 complex. Cargo is recognized by recognition molecules such as p62/SQSTM1, which bind ubiquitinated substrates and LC3-II in the autophagosome membrane. Mature autophagosomes then fuse with lysosomes through Rab7, snare, and hops complexes, forming autolysosomes where macromolecules are degraded and recycled into amino acids, lipids, and nucleotides.

Autophagy is an evolutionarily conserved lysosomal degradation pathway that maintains cellular homeostasis by recycling biosynthetic substrates – including organelles, misfolded proteins, and cytoplasmic macromolecules – during periods of nutrient deprivation and cellular stress to support metabolic compensation and cell survival. The process classically begins with the ULK1 complex (ULK1/2, ATG13, FIP200, ATG101), which integrates nutrient and stress cues from upstream signaling molecules, including mTORC1 and AMPK [1,2], triggering the formation of the PI3K complex (Beclin-1, VPS34, VPS15, ATG14L) and nucleation of the phagophore membrane [3,4] (Figure 1). Elongation and maturation of the budding autophagosome membrane require the ATG12–ATG5–ATG16L1 conjugation system and lipidation of the ATG8 family of proteins, a process that is critically dependent on ATG7, an E1-like enzyme that initiates the conjugation cascade. While LC3 is the most thoroughly characterized and frequently studied ATG8 family member, it is only one of seven mammalian ATG8 homologs that exhibit significant functional overlap [5–7]. The redundancy of these homologs underscores the importance of autophagy as a conserved cellular process, yet how they direct specific autophagic processes or compensate for one another under disease-specific stress conditions remains poorly understood. Upon lipidation, the ATG8 family of proteins anchor cargo adaptor proteins, such as p62 and SQSTM1 [8,9], to facilitate selective degradation. Finally, the autophagosome fuses with the lysosome via coordinated mediators, including Rab7, LAMP2, and SNARE proteins [10–12], to form the autolysosome, wherein macromolecules are degraded into their constituent monomers. This dynamic and highly regulated process is essential for the maintenance of cellular redox balance, proteostasis, and organelle quality control, and its dysregulation has been implicated in numerous human diseases, including cancer, neurodegeneration, and metabolic disorders [13]. The combined biosynthetic demands of the endocrine and exocrine pancreas create a unique proteostatic lens through which to analyze the role of autophagic flux on maintaining the integrity of secretory cells.

**Figure 2.**
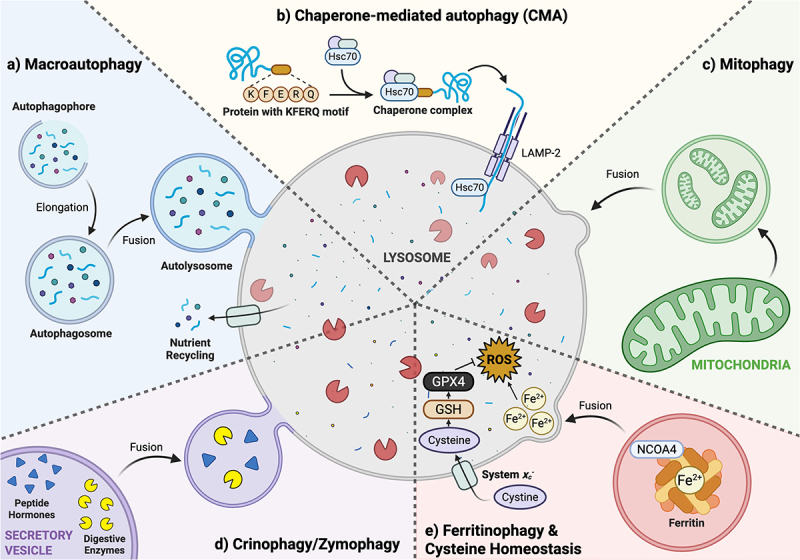
Major forms of autophagy in pancreatic physiology.(a) Macroautophagy: non-selective sequestration of cytoplasmic cargo into double membrane autophagosomes, which subsequently fuse with lysosomes to form autolysosomes for cargo degradation and intracellular recycling. (b) Chaperone-mediated autophagy (CMA): selective translocation of cytosolic proteins containing a KFERQ-like motif directly across the lysosomal membrane via the Hsc70 chaperone and LAMP-2A receptor. (c) Mitophagy: selective autophagy of damaged or dysfunctional mitochondria to maintain mitochondrial quality control and metabolic homeostasis. (d) Crinophagy/Zymophagy: a specialized pathway in pancreatic acinar and endocrine cells involving direct fusion of secretory granules (e.g., digestive enzymes or peptide hormones) with lysosomes for degradation. (e) Ferritinophagy: selective NCOA4-mediated ferritin degradation and system *x_c_*^−^ driven glutathione synthesis, which together regulate the labile iron pool and redox homeostasis to prevent iron-dependent ROS generation.

The pancreas is among the most functionally diverse organ systems in the human body, producing enzymes critical for digestive processes and hormones necessary for systemic metabolic and glucose homeostasis. Due to the exceptionally high levels of protein synthesis, secretion, and turnover, pancreatic secretory cells are uniquely dependent on efficient mechanisms of peptide quality control. This balance is maintained through both ubiquitin-proteosome-mediated degradation and various autophagic mechanisms. Autophagy in the pancreas can take multiple specialized forms depending on the substrates sequestered for degradation. Macroautophagy (hereafter referred to as “autophagy”) is a relatively nonselective process that mediates the bulk degradation of cytoplasmic material and aberrant protein aggregates. Other forms of autophagy include chaperone-mediated autophagy (CMA), which selectively degrades specific molecules harboring recognition motifs; mitophagy, which recycles damaged mitochondria; crinophagy, which directs hormone-containing secretory granules to lysosomal degradation; and zymophagy, a process unique to the exocrine pancreas that eliminates prematurely activated zymogen granules (Figure 2). This review will explore the role of these autophagic pathways in pancreatic development, normal physiological function, and their dysregulation in disease states.

## Autophagy in pancreatic development and homeostasis

Autophagy has a well-defined and increasingly recognized role in maintaining pancreatic homeostasis under physiological conditions. In both the exocrine and endocrine pancreas – as well as in pancreatic embryonic development – basal autophagy functions to maintain metabolic energy balance, regulate organelle turnover, and mitigate intracellular stress. By sustaining autophagic flux, the pancreas is able to support tissue maturation, the regulated release of peptide hormones and digestive enzymes, and cellular resilience to fluctuations in nutrient availability and metabolic demand.

There is accumulating evidence supporting a critical role for autophagy in pancreatic embryonic and early post-natal development. For example, global deletion of core autophagy genes (e.g., *Atg5* and *Atg7)* results in severe developmental phenotypes: mice lacking *Atg5* die within hours of birth, unable to survive the post-natal starvation period during which autophagy is required to mobilize amino acids and maintain energy output [14]. Similarly, *Atg7*^−/−^ mice succumb during the neonatal period due to a combination of energy depletion, accumulation of abnormal organelles, and widespread tissue degeneration [15]. In contrast, pancreas-specific conditional knockouts of *Atg5* or *Atg7* are viable but predisposed to a severe pancreatitis phenotype mediated by prematurely activated trypsin and resulting in spontaneous chronic atrophic pancreatitis with acinar degeneration, inflammation, ADM, fibrosis, and pancreatic atrophy [16,17]. More recently, conditional, pancreas-specific knockout of *Beclin1*, a key regulator of autophagosome initiation and nucleation, was shown to result in pancreatic agenesis and severe defects in the endocrine and exocrine compartments [18]. Together, these data suggest that autophagic machinery is essential for pancreatic organogenesis and that disruption of key *Atg* genes impairs normal maturation, underscoring its developmental importance.

The exocrine pancreas, primarily composed of acinar cells, is responsible for the synthesis and secretion of digestive enzymes and relies heavily on autophagy to maintain cellular integrity. In acinar cells, autophagy regulates enzyme granule turnover, alleviates oxidative stress, and maintains protein quality control to prevent cellular injury. In fact, the mammalian exocrine pancreas exhibits one of the highest levels of basal autophagy among all organ systems – exceeding that of the liver, kidney, heart, and endocrine pancreas [19] – underscoring the key role of autophagy in maintaining acinar cell homeostasis.

Basal autophagy functions to clear misfolded or surplus digestive enzyme precursors and defective zymogen granules, preventing their premature intra-acinar activation. This process becomes especially critical in the fed state, when increased secretory demand can overwhelm the ER and heighten the risk for proteotoxic stress [20,21]. The surge of enzyme synthesis can overload the ER, leading to the accumulation of misfolded zymogens and increasing the likelihood of premature activation [22]. Under normal circumstances, compensatory autophagy and ER-stress pathways (including the unfolded protein response) are sufficient to restore proteostasis and protect acinar cells against toxic effects; however, when these mechanisms become overwhelmed or impaired, acinar cell destruction and disease can ensue [22]. Indeed, pancreas-specific disruption of essential autophagy genes *Atg5* or *Atg7* (*Pdx1*- or acinar-Cre) results in accumulation of p62, increased ER and mitochondrial stress, and progressive pancreatic injury characterized by distortion of pancreatic architecture, chronic pancreatitis, and acinar-to-ductal metaplasia, demonstrating that basal autophagy is indispensable for exocrine homeostasis [21]. Notably, the persistence of a macroscopic pancreatic organ in these models suggests that while *Atg5/7*-mediated conjugation is indispensable for the functional and structural maturation of the pancreas, it is not strictly required for initial lineage specification [17,21]. This is in contrast to the more foundational role of upstream autophagy initiators such as Beclin1, the loss of which results in complete pancreatic agenesis [18]. This stark phenotypic disparity may stem from diverse scaffolding roles Beclin1 plays in parallel signaling pathways (e.g., PI3K complex [23,24]), or suggest that *Atg5/7*-independent, non-canonical autophagy is sufficient to drive specification of early pancreatic progenitor tissues [25,26]. Thus, the autophagy machinery likely functions as a stage-specific mediator rather than a binary requirement in pancreas organogenesis and maturation.

The primary role of the endocrine pancreas is the maintenance of blood glucose through the release of hormones that regulate carbohydrate, lipid, and protein metabolism [27,28]. The endocrine pancreas is composed of a-, b-, and d-cells, which secrete glucagon, insulin, and somatostatin, respectively, and cooperate to maintain systemic energy balance. β-cells, which account for approximately 80% of the endocrine pancreas mass, play the dominant role in maintaining glucose homeostasis through the synthesis and secretion of insulin. As the body’s master anabolic signaling hormone [29], insulin production and secretion are tightly regulated processes, and even minor perturbations can profoundly affect systemic glucose uptake and energy metabolism.

Within β-cells, autophagy is essential for maintaining insulin secretion and β-cell mass; it modulates insulin granule turnover, prevents the accumulation of misfolded peptides, and safeguards cells from stress-induced apoptosis [30,31]. The β-cell must be able to rapidly ramp up production of insulin in response to external stimuli, particularly in the fed state. This requirement makes β-cells uniquely vulnerable to endoplasmic reticulum (ER) stress [32], with any disturbances in ER-mediated protein synthesis putting β-cells at risk of dysfunction and apoptosis [33,34]. Recent studies indicate that autophagy can compensate for an overwhelmed ER, functioning to degrade misfolded proteins and prevent damage from proteotoxic and oxidative stressors [35,36]. Loss of core autophagy genes, including *Atg5* and *Atg7*, displays impaired insulin granule formation and secretion, ultimately resulting in reduced β-cell mass and impaired glucose tolerance [31]. Conversely, pharmacologic and genetic augmentation of autophagy through mTORC1 modulation improves β-cell integrity and insulin secretion in preclinical models of diabetes [37,38], underscoring autophagy as a critical adaptive signaling pathway safeguarding β-cell function and preserving systemic metabolic homeostasis.

## The role of autophagy in diseases of the pancreas

Due to its prominent role in maintaining pancreatic homeostasis, it follows that dysregulation of autophagic signaling can drive cellular injury and disease. When autophagy is disrupted, processes that normally function to alleviate stress can become maladaptive and contribute to cellular dysfunction, impaired secretion, inflammation, and oxidative stress. As such, autophagic deficits have been described in various diseases of the pancreas, including pancreatitis, diabetes mellitus, and pancreatic neoplasms. Therefore, further understanding of the biologic underpinnings of the balance between protective and pathogenic autophagy not only provides crucial insight into disease mechanisms but also provides a novel window for therapeutic leverage.

### Pancreatitis

Pancreatitis is a pathological inflammation of the pancreatic tissues, defined histologically by chronic or acute necrosis of pancreatic acinar cells, resulting in localized or systemic inflammation [39]. The pathophysiology of pancreatitis is classically thought to be due to premature trypsin activation within pancreatic acinar cells with subsequent “auto-digestion” of pancreatic tissues [40,41]. Mounting evidence suggests autophagy plays a critical role in protecting against pancreatitis, whereby impaired autophagy-mediated processing of digestive enzymes leads to the intracellular conversion of trypsinogen to trypsin, acinar cell death, and spontaneous pancreatitis. Here, we discuss the current understanding of the role of autophagy in pancreatitis pathogenesis.

Pancreatitis often displays features of lysosome disorders and vacuolization. Large intracellular vacuoles are a long-standing histological hallmark of pancreatitis, and recently these vacuoles have been shown to stain positively for LC3 and LAMP1 [42], directly linking defective autophagosome formation and maturation to pancreatic injury. Indeed, pancreas-specific deletion of core autophagy genes, including *Atg5* and *Atg7*, as well as global knockout of *Lamp2*, predisposes mice to spontaneous pancreatitis [17,43,44] – providing definitive evidence that intact autophagy is critical for acinar homeostasis. Moreover, in murine models of pancreatitis, fasting-induced autophagy increases Beclin-1 levels, lowers serum amylase, reduces histologic damage, and mitigates disease severity [45], further supporting the role of autophagic flux as a protective mechanism against injury. Furthermore, it has been shown that autophagosome maturation and fusion with lysosomes become impaired in pancreatitis, compromising activation of lysosomal proteases cathepsins B and L and reducing enzymatic activity in lysosome-enriched fractions [46], altogether suggesting autophagy is activated in pancreatitis, but autolysosome formation and function are defective.

Despite this evidence, the precise role of autophagy in pancreatitis remains complex and somewhat context-dependent. Initial studies using conditional *Atg5* knockout in acinar cells demonstrated *reduced* caerulein-induced pancreatitis and diminished intracellular trypsin activation [16], suggesting a deleterious role for autophagy under specific stress conditions. This was supported by subsequent experiments in which overexpression of GFP-LC3, a marker of enhanced autophagosome formation, exacerbated caerulein-induced injury [46,47]. However, more recent work indicates that the loss of *Munc18c* increases autophagic flux and reduces pancreatitis disease severity [48]. These seemingly conflicting observations can be reconciled by distinguishing bulk autophagy from zymophagy – the selective autophagic degradation of activated zymogen granules – in regulating exocytosis. Indeed, loss-of-function experiments with *Irf2*, *Munc18c*, *Snap23*, and *Vamp8* – all regulators of exocytosis – attenuate caerulein-induced pancreatitis [48–51], whereas disruption of proteins involved in vesicular fusion and lysosomal biogenesis (e.g., *Vmp1*, *Stx17*, *Tfeb*, or *Tfe3*) worsens pancreatic injury [52–54]. These data suggest that preventing autophagosome formation (e.g., via *Atg5* deletion) may increase the relative availability of lysosomes to fuse directly with zymogen granules, thereby promoting more efficient degradation of the activated zymogens responsible for inciting pancreatitis.

Meanwhile, growing evidence indicates that dysregulated protein synthesis and folding may contribute to aberrant zymogen activation in pancreatitis. The exocrine pancreas synthesizes and secretes digestive enzymes as inactive zymogens in abundance, a process that demands precise coordination between the endoplasmic reticulum (ER), Golgi, and mitochondria [20]. Disruption of this system can provoke ER stress and mitochondrial dysfunction, and further impair autophagic flux. For example, VMP1, an ER membrane protein, has recently emerged as a key protective factor against ER stress. Acinar-specific deletion of VMP1 induces ER stress, impairs autophagic degradation, and recapitulates the histologic features of acute pancreatitis [52]. Similarly, pancreatitis-induced opening of the mitochondrial permeability transition pore (MPTP) has been shown to disrupt autophagic signaling, increasing zymogen activation and acinar cell necrosis, effects that can be reversed by pharmacologic inhibition of the MPTP [55]. Impairment of ATP synthase activity has likewise been shown to exacerbate ER stress and suppress autophagic flux, culminating in premature activation of zymogens [56]. Together, these findings emphasize that crosstalk between the ER, mitochondria, and lysosomes is critical for acinar cell fitness and highlight defective autolysosome formation as a central pathogenic feature of pancreatitis.

Finally, among the various inciting factors of pancreatitis, the most clinically prominent is excessive alcohol (ethanol) intake. Alcohol-induced pancreatitis is an untreatable sequela of chronic alcohol consumption that currently accounts for up to 25% of all acute pancreatitis cases [39]. Although mechanistic details remain incompletely defined, growing evidence suggests ethanol exposure can disrupt multiple stages of autolysosome formation within pancreatic acinar cells. Recent studies show that alcohol upregulates the cysteine protease ATG4B, which regulates the balance of cytosolic LC3-I and membrane-bound LC3-II. Elevated ATG4B activity depletes conjugated LC3-II and impairs autophagosome maturation, thereby contributing to the cellular injury in alcoholic pancreatitis [57]. In parallel, alcohol has been shown to suppress the activity of the transcription factor TFEB, a master regulator of autophagy and lysosomal biogenesis, through both transcriptional and post-transcriptional mechanisms [54,58]. Moreover, both acute and chronic alcohol exposures have been shown to activate different components of the unfolded protein response (UPR) – including GRP78, p-IRE1α, XBP1, and CHOP [59] – reflecting sustained ER stress and further compromising protein homeostasis. These findings provide new insight into the mechanisms by which ethanol exposure simultaneously impairs autophagosome maturation, lysosomal biogenesis, and ER homeostasis and predisposes to pancreas injury.

### Diabetes mellitus

Diabetes Mellitus (DM) is a chronic metabolic disorder secondary to an insufficient response to insulin signaling, leading to decreased glucose uptake and increased blood glucose levels. The two most prevalent forms of DM are type 1 diabetes mellitus (T1DM), characterized by insufficient insulin production due to autoimmune β-cell destruction, and type 2 diabetes mellitus (T2DM), characterized by insulin resistance and β-cell dysfunction [60,61]. Diabetes mellitus imposes a significant physiologic stress on pancreatic β-cells, the insulin-producing cells of the endocrine pancreas. To combat this stress, β-cells rely on autophagy to alleviate ER stress and the unfolded protein response (UPR), mitigate oxidative stress due to excess reactive oxygen species (ROS), and suppress inflammation through inactivation of the NLPR3 inflammasome complex [62]. Here, we summarize recent insights into how autophagy dysregulation contributes to the onset and progression of diabetes mellitus.

#### Type 1 diabetes: cellular destruction

Type 1 diabetes mellitus (T1DM) is characterized by immune-mediated destruction of pancreatic β-cells, resulting in an absence or relative deficiency of insulin and chronic hyperglycemia [63]. Reduced autophagosome-lysosome colocalization and accumulation of autophagic vacuoles within β-cells has been a long-observed phenomenon in pancreatic tissue of patients with T1DM^64^. Subsequent complementary studies utilizing the non-obese diabetic (NOD) mouse model (a model of spontaneous autoimmune diabetes) demonstrated that b-cell specific deletion of key autophagic components such as *Atg7* precipitated hyperglycemia with reduced insulin secretion [31], providing early evidence for a protective role of autophagy in T1DM. More recent studies have focused on the role of crinophagy, the selective fusion of insulin-containing secretory vesicles with lysosomes for degradation. In diabetic NOD mice, proinsulin content has been shown to be decreased in the lysosomes of diabetic NOD mice, accompanied by an accumulation of telolysosomes with undigested material [64]. Notably, these defects precede overt hyperglycemia, suggesting that autophagic impairment may be an early predisposing event contributing to β-cell failure and hypoinsulinemia.

The pathogenesis of T1DM is thought to be due to infiltration of auto-reactive cytotoxic T cells into pancreatic islets with subsequent destruction of a β-cell-expressed autoantigen [65]. It therefore follows that antigen presentation by MHC class I (HLA-I) molecules on the surface of pancreatic β-cells and intracellular protein degradation pathways would play a key role in T1DM pathogenesis. Additionally, immune-mediated destruction and release of autoantigen into the pancreatic microenvironment and cross-presentation by antigen-presenting cells (APCs), such as dendritic cells, would function to induce further inflammation and β-cell destruction. Autophagy has been implicated in both processes. Recent work has shown that autophagy inhibition increases MHC-I/HLA-I presentation to surveilling immune cells. In particular, inhibition of autophagic flux with Bafilomycin A1 exacerbates the surface expression of MHC-I, whereas stimulation of lysosomal activity via transcriptional activation of TFEB enhances degradation and reduces surface MHC-I levels [66]. Meanwhile, autophagy has been shown to be indispensable for dendritic cell-mediated autoimmune responses, with the loss of autophagy in this cell type resulting in a failure to prime autoreactive CD4 T cells [67]. Taken together, these findings highlight a multifaceted role for autophagy in regulating β-cell homeostasis and suggest dysregulated autophagy may contribute to both the intrinsic β-cell dysfunction and the aberrant immune activation underlying T1DM pathogenesis.

#### Type 2 diabetes: metabolic stress

From a clinical perspective, Type 2 diabetes mellitus (T2DM) is a far more prevalent and chronically progressive metabolic disorder, affecting as many as 800 million people worldwide [68]. T2DM is broadly defined by systemic resistance to insulin signaling in peripheral tissues coupled with impaired insulin secretion from dysfunctional β-cells, leading to chronic hyperglycemia [69]. Human pancreatic tissues from patients with T2DM have consistently shown an accumulation of autophagosomes within pancreatic β-cells, suggesting defective autophagic clearance and implicating impaired autophagy in disease progression. Further, pancreatic islets of patients with T2DM show features consistent with impaired autophagic flux, including increased LC3 foci, p62 accumulation, and decreased lysosomal LAMP2 [62,70,71], providing a strong rationale for the exploration of autophagy as a mediator of diabetes.

To begin interrogating the relationship between autophagy and T2DM, several studies employed conditional knockout models targeting autophagy genes in pancreatic β-cells. For example, β-cell-specific deletion of *Atg7* has been shown to recapitulate hallmark signs of T2DM, including reduced circulating insulin, β-cell dysfunction, and accumulation of intracellular protein aggregates and damaged organelles [31,72], leading to β-cell dysfunction and death. In humans, the principal driver of T2DM is a diet-related surplus of glucose and free fatty acids (FFAs), increasing demand for insulin production and leading to increased oxidative stress and ER toxicity [73–75]. Consistent with this, mice fed a high-fat diet exhibit increased autophagic demand, as evidenced by the accumulation of autophagosomes [76]. Further studies using *Atg7*-deficient mice demonstrated that a high-fat diet in the setting of autophagic dysfunction results in classical clinical features of T2DM, including β-cell death, providing a mechanistic link between autophagy and predisposing factors for human disease [72]. This fundamental requirement for autophagic machinery is underscored by the recent identification of deleterious *ATG7* mutations in human patients. These affected individuals exhibit a complex neurodevelopmental syndrome and multi-organ dysfunction, confirming that although some downstream components of the autophagy pathway exhibit redundancy (e.g., ATG8 family members), ATG7 serves as an essential, non-redundant bottleneck for human cellular homeostasis.

While macroautophagy is classically induced by nutrient deprivation and degrades cytosolic components in a relatively indiscriminate manner, T2DM is a disease of nutrient excess and relies on the regulation of a specific peptide, namely insulin. It therefore follows that other forms of autophagy may play distinct roles in β-cell maintenance. Indeed, studies utilizing Rab-deficient mouse models to mimic the secretory defects seen in T2DM indicate that crinophagy can act as a compensatory mechanism when autophagy is impaired, maintaining insulin turnover and β-cell integrity [77]. Moreover, chronic inflammation of pancreatic islets is a well-characterized sequela of T2DM and contributes to β-cell damage and death. Recent work identified CLEC16A, a key regulator of mitophagy, as a key protective factor in this context. Overexpression of CLEC16A alleviated cytokine- and inflammation-induced damage of pancreatic β-cells, whereas its deletion exacerbated hyperglycemia and β-cell apoptosis [78]. These findings underscore the importance of mitochondrial quality control via mitophagy in preserving β-cell viability and suggest that defects in selective autophagy pathways – both crinophagy and mitophagy – may converge to drive β-cell dysfunction in T2DM.

Finally, dietary patterns are currently an active area of investigation in the progression and prevention of T2DM. It is now more broadly accepted that intermittent fasting – an umbrella term that includes time-restricted feeding (TRF; e.g., 6–10-h daily eating windows), alternate-day fasting, and 5:2 regimens – has been associated with weight loss and modest improvements in glycemic control in overweight or prediabetic adults [79,80]. Notably, in a randomized study of a time-restricted feeding (TRF) diet in men with prediabetes, insulin levels, insulin sensitivity, β cell responsiveness, blood pressure, and oxidative stress levels were all improved despite matched caloric intake and no significant weight loss [81], suggesting a weight-independent improvement of fasting on glycemic control. These findings have now been substantiated with preclinical work: it has recently been shown in a non-obese, insulin secretory-deficient, K_ATP_-induced mouse model of diabetes that intermittent fasting restores both autophagic flux and β-cell mass in the pancreatic islets when compared to mice given a continuous diet [82]. These results should motivate the initiation of further trials to delineate which fasting schedules offer the most benefit on HbA1c and β-cell function in patients with T2DM.

While autophagy is generally regarded as protective against the initiation and progression of diabetes, emerging evidence suggests that excessive autophagic flux can also compromise β-cell health and integrity. For instance, crinophagy-mediated degradation of insulin granules has been proposed to transform insulin from an inert thymic self-antigen into a reactive autoantigen by narrowing the insulin-based epitope repertoire in pancreatic islets of patients with T1DM [83]. Moreover, under physiologic conditions, autophagic recycling of mitochondria (mitophagy) mitigates oxidative stress and restricts inflammasome activation; however, excessive or sustained autophagy may trigger autophagic cell death when persistently activated [84]. Indeed, autophagy-driven activation of the NLRP3 inflammasome complex has been shown to promote a localized inflammatory response and exacerbate β-cell damage in both type 1 and type 2 diabetes mellitus [85]. These findings highlight the context-dependent nature of autophagy in diabetes and underscore the need for further studies to determine the therapeutic boundaries between its protective and pathogenic roles.

### Pancreatic adenocarcinoma (PDAC)

Pancreatic adenocarcinoma (PDAC), the most common malignancy of the pancreas, is among the deadliest of all human cancer types. Characterized by aggressive local invasion, early metastatic dissemination, and a profound resistance to conventional chemotherapy, PDAC carries a 5-year overall survival of 13% [86]. A defining feature of PDAC is its dense desmoplastic stroma and hypovascular, hypoxic tumor microenvironment, which together restrict nutrient and oxygen delivery and create a metabolically hostile environment for cellular survival. To survive these stressors, PDAC cells reprogram their internal metabolic pathways to maintain energy output and biosynthetic flux using adaptive mechanisms such as autophagy [87,88]. Although initially described as tumor suppressive [89], autophagy is now accepted as a critical mediator of PDAC tumorigenesis, supporting cellular growth in nutrient-depleted conditions and conferring resistance to chemotherapy, immunotherapy, and targeted agents [87]. Here, we summarize current mechanistic insights into how autophagy supports PDAC initiation, progression, and survival.

#### Role in rumor initiation and maintenance

Early studies exploring autophagy in oncogenesis found that PDAC cells display a markedly elevated basal autophagy relative to tumor cell types [90], suggesting a unique role for autophagic flux in the initiation and progression of pancreatic tumors. Given that more than 90% of pancreatic tumors harbor a KRAS-activating mutation leading to constitutive MAPK pathway activation [91], this observation led to the hypothesis that elevated autophagic flux in PDAC is KRAS-dependent and required for tumor maintenance. Indeed, early studies found that continuous autophagy inhibition induces extensive pancreatic metaplasia, but only in the presence of an oncogenic KRAS mutation [92]. Furthermore, intermittent autophagy inhibition in KRAS-mutant pancreata also failed to induce metaplasia, implying that sustained loss of autophagy is required to cooperate with oncogenic KRAS during early tumorigenesis – yet dispensable for malignant transformation in normal tissue. These studies provided important biological insight into the interplay between mutant KRAS, autophagy, and PDAC disease progression.

To directly address the dependency of PDAC tumor progression on autophagy, Yang and colleagues established a genetically engineered mouse model of spontaneous PDAC (*Pdx1*^*Cre+*^, *LSL-Kras*^*G12D/+*^, *Trp53*^*lox/+*^) combined with either heterozygous or homozygous deletion of the essential autophagy gene *Atg5*. Loss of *Atg5* effectively suppressed autophagic flux in tumor cells and resulted in the accumulation of pancreatic intraepithelial neoplasms (PanINs) – a precursor lesion to PDAC – but did not progress to invasive carcinoma in the context of *Trp53* heterozygosity [93]. Subsequent studies from the same group utilized an additional mouse model of PDAC (*LSL-Kras*^*G12D*^, *Trp53*^*lox/+*^, *p48*^*Cre+*^) featuring doxycycline-inducible expression of a dominant-negative Atg4b construct (*Atg4b*^*C74A*^). This mutant prevents LC3 lipidation and inhibits autophagosome membrane formation, providing a model wherein autophagy can be readily and reversibly inhibited in tumor cells [94]. Using this pancreas-specific expression, the authors demonstrated that potent autophagy inhibition through homozygous expression of the dominant-negative *Atg4b* (*Atg4b*^*C74A*^) resulted in significant tumor regression in established PDAC tumors. Notably, this regression was driven by both cell-intrinsic and cell-extrinsic mechanisms, as global *Atg4b*^*C74A*^ expression reduced the availability of host-derived amino acids necessary for tumor growth. Furthermore, the authors observed a strong selective pressure for autophagic flux as mice heterozygous for *Atg4b*^*C74A*^ frequently lost the mutant allele, leading to restored autophagy and tumor recurrence [94]. These findings were ultimately corroborated by pharmacologic inhibition using chloroquine or hydroxychloroquine in murine PDAC cell lines and patient-derived xenografts, which reduced proliferation and increased apoptosis, collectively suggesting a critical dependence on autophagy for PDAC tumor maintenance.

#### Autophagy as a mediator of therapeutic resistance

The role of autophagy becomes especially pertinent in the context of anti-cancer treatment. Autophagy has emerged as a resistance mechanism that allows for cell persistence in the face of chemotherapy-induced stress signals, functioning to turn over damaged organelles and repair important signaling programs following chemo- and radiotherapy [95,96]. For example, inhibition of the RAS→ RAF→ MEK→ ERK MAPK signaling pathway using Trametinib has been shown to activate the LKB1→ AMPK→ ULK1 signaling axis, resulting in activation of autophagy as an escape mechanism to the cytotoxic effects of treatment [97]. Such protective autophagic mechanisms have also been shown to be induced following direct ERK inhibition [98], as well as with the use of EGFR inhibitors gefitinib and erlotinib [99], leading to AMPK activation and decreased mTORC1 signaling, two well-characterized drivers of autophagy. Further, autophagy has recently emerged as a resistance mechanism to immunotherapy and the evasion of immune surveillance [100,101]. Yamamoto et al. demonstrated that the activation of autophagy leads to selective internalization and degradation of MHC-I molecules in PDAC cells. Notably, chloroquine-mediated inhibition of autophagy restored surface expression of MHC-I, increased CD8 T cell-mediated cancer cell killing, and synergized with immune-checkpoint blockade (anti-PD1 and anti-CTLA4) [100].

It should be noted that while emerging evidence indicates that autophagy supports tumor initiation and the progression of early precancerous lesions into malignant PDAC, its loss in established tumors may instead promote epithelial – mesenchymal transition (EMT) and enhanced invasion [102]. In a study by Wang et al., knockdown of *Atg5*, a core autophagy gene, led to an upregulation of pro-EMT markers, including ZEB1 and SNAIL, and resulted in increased migration and invasion in PDAC models [103]. Similarly, Görgülü et al. reported that while homozygous deletion of *Atg5* abrogated PDAC tumorigenesis, heterozygous deletion was associated with an invasive, immunosuppressive tumor microenvironment and increased metastatic spread *in vivo* relative to wildtype control [104], suggesting that whether autophagy exerts a pro- or anti-tumorigenic effect may be influenced by the stage of disease, underlying genetic alterations, and the surrounding tumor microenvironment.

#### Ferritinophagy and redox balance

While bulk autophagy remains the primary driver of nutrient scavenging and recycling in PDAC, recent progress has distinguished specialized selective pathways as essential for the recovery of specific metabolic precursors. Foremost among these pathways is ferritinophagy, or the selective autophagic degradation of ferritin, which has emerged as a critical mediator of pancreatic cancer progression. The requirement for ferritinophagy was underscored by two landmark studies demonstrating that PDAC cells leverage this pathway to increase the bioavailability of iron, thereby bolstering mitochondrial fitness. Santana-Codina et al. demonstrated that PDAC exhibits a significant upregulation of NCOA4, an autophagy cargo receptor that recruits ferritin into elongating autophagosomes. Upon lysosomal degradation of ferritin, the resulting increase in the intracellular iron pool drives the synthesis of iron-sulfur clusters (ISCs), which are essential for maintaining mitochondrial homeostasis and oxidative phosphorylation. Accordingly, genetic deletion of *Ncoa4* was shown to delay tumor growth and progression in murine models of PDAC [105]. An associated study from Ravichandran et al. revealed that ferritinophagy can be further activated in response to therapeutic stress. MAPK inhibition, which induces a metabolic crisis and activates autophagy, was found to promote ferritinophagy, leading to the accumulation of ferritin and transferrin within the lysosomal compartment of MEKi-treated cells. Mechanistically, the suppression of MAPK signaling reduced the stability of c-Myc, thereby allowing increased access of other master transcription factors, including TFE3 and TFEB, to bind genomic DNA and drive the coordinated lysosomal expression and regulation (CLEAR) network to upregulate lysosomal biogenesis [106]. This coordinated transcriptional program enhances mitochondrial oxidative phosphorylation and tricarboxylic acid (TCA) cycling, providing the metabolic framework necessary to withstand MEK inhibition.

While ferritinophagy represents a potential adaptive bypass pathway to therapeutic intervention in PDAC [107], the mobilization of free iron functions as a “double-edged” sword. Under normal circumstances, PDAC cells rely on the System *x*_*c*_^−^ transporter to import extracellular cystine. Once internalized, cystine is reduced to cysteine for the synthesis of glutathione (GSH), an essential cofactor for glutathione peroxidase 4 (GPX4), which neutralizes reactive oxygen species (ROS) and prevents iron-dependent lipid peroxidation [108,109]. Consequently, iron and cysteine homeostasis are intrinsically linked through the GPX4-regulated redox axis. Indeed, emerging evidence suggests that excessive iron accumulation can overwhelm these intracellular antioxidant systems, leading to the generation of reactive oxygen species (ROS), lipid peroxidation, and ferroptotic cell death. Multiple studies have demonstrated that hyperactivating NCOA4 – either through ATM-mediated phosphorylation or loss of E3-ligation-mediated degradation – drives constitutive, excessive ferritinophagy resulting in an expansion of the labile iron pool (LIP) and lethal ferroptosis [110–112].

From a translational perspective, these results illustrate a therapeutic dichotomy: pharmacologic intervention can be designed to either inhibit ferritinophagy below the basal level required for tumor growth and metabolic fitness, or, conversely, to hyper-activate the NCOA4-ferritinophagy axis to drive cells past their lethal ferroptosis threshold [113]. By using tools such as Ferroptosis-Related Gene (FRG) signatures in patient cohorts [114,115], clinicians may eventually be able to stratify PDAC patients by their basal iron burden and redox state, moving beyond observational correlation into targeted, iron-modulating treatments.

## Therapeutic implications

Due to the growing role of autophagy in pancreatic physiology and disease progression, there is an increased interest in therapeutically targeting multiple stages of the autophagy signaling pathway. Depending on the disease context, autophagy serves as a metabolic dependency that sustains tumor growth (as in PDAC) or as a protective mechanism against inflammatory or proteotoxic stressors (as in diabetes and pancreatitis). This context-dependent duality underscores the dichotomous nature of autophagic signaling, wherein inhibition can sensitize malignant cells to therapy while activation may preserve normal tissue integrity under stress conditions. Here, we summarize current preclinical and clinical advances in autophagy-modulating strategies (and novel combinations therein) and discuss their therapeutic implication across pancreatic disease states.

### Autophagy inhibition in anti-oncologic treatment

#### First-generation lysosomotropic agents

**Figure 3.**
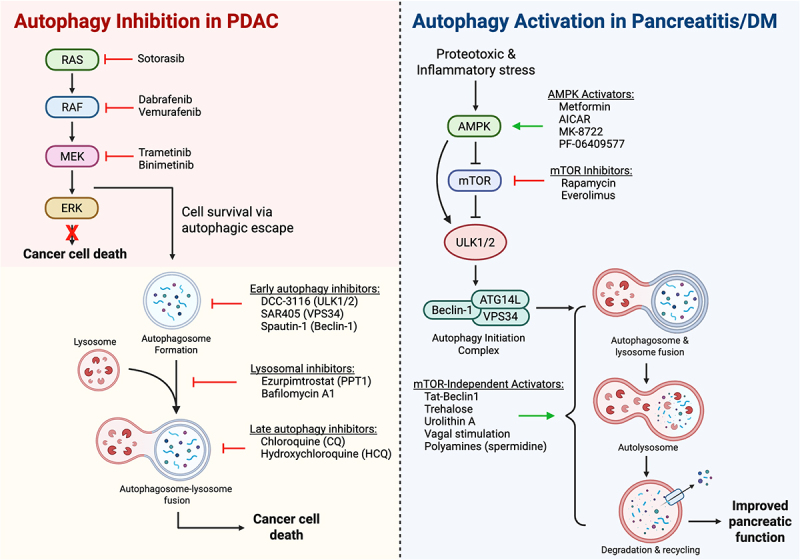
Effect of autophagy modulation in pancreatic pathology, including pancreatic ductal adenocarcinoma (PDAC) versus pancreatitis/diabetes mellitus (Dm).(left) in pancreatic ductal adenocarcinoma (PDAC), inhibition of the MAPK pathway (RAS, RAF, MEK) induces compensatory autophagy that supports tumor survival under therapeutic stress. Early-stage autophagy inhibitors (ULK1/2, VPS34, Beclin-1 modulators) and late-stage/lysosomal inhibitors (PPT1 inhibitors, bafilomycin A1, chloroquine derivatives) enhance the efficacy of MAPK inhibition by restricting metabolic plasticity and stress adaptation, resulting in increased cancer cell death. (right) in pancreatitis and diabetes mellitus (DM), impaired autophagic clearance contributes to proteotoxic and inflammatory stress and β-cell dysfunction. AMPK activators (metformin, AICAR, MK-8722) or mTOR inhibitors (rapamycin, everolimus) promote ULK1-dependent autophagy initiation, restoring cellular homeostasis. mTOR-independent inducers (Tat-Beclin-1, trehalose, urolithin a, vagal stimulation, polyamines), have also shown preclinical evidence of enhanced autophagic flux, improving β-cell function and pancreatic tissue homeostasis.

Since the discovery that autophagy is constitutively elevated and required for tumor growth in pancreatic ductal adenocarcinoma [90], targeted inhibition of the autophagic signaling axis has emerged as an attractive therapeutic strategy (Figure 3). The first autophagy inhibitors explored clinically came in the form of chloroquine (CQ) and its derivative hydroxychloroquine (HCQ) [116]. Originally developed as anti-malarial agents, these compounds were repurposed as anti-autophagic cancer therapeutics due to their lysosomotropic properties, which allow them to accumulate in lysosomal vesicles and disrupt late-stage autophagy by inhibiting autophagosome-lysosome fusion and cargo degradation [116]. Early proof-of-concept evidence for these inhibitors came from a randomized, double-blind trial in patients with glioblastoma multiforme (GBM) wherein the addition of chloroquine to standard chemoradiation improved median overall survival (OS) from 11 to 24 months [117] (NCT02432417), establishing a clinical precedent for further exploration in other autophagy-dependent malignancies such as PDAC.

The first PDAC trial to evaluate this approach – a randomized phase II study of gemcitabine/nab-paclitaxel ± HCQ in patients with metastatic PDAC (MAPS; NCT01506973) – demonstrated no improvement in 12-month OS, though the objective response rate (ORR) increased from 21% to 38% with the addition of HCQ [118]. A subsequent randomized phase II neoadjuvant trial using high-dose HCQ with gemcitabine/nab-paclitaxel similarly failed to improve survival outcomes but reported enhanced pathologic and biologic response metrics before surgery [119] (PACS; NCT01978184). Building on these findings, a prospective neoadjuvant study exploring the long-term effects of HCQ in 35 patients with resectable PDAC found that preoperative HCQ plus gemcitabine yielded a median OS of 31 months following resection [120], markedly longer than the historical OS of ~15 months with gemcitabine alone [121]. Notably, 48% of HCQ-treated patients demonstrated histopathological responses at the time of resection, and this cohort achieved an overall survival approaching 5 years [120], an exceptional metric in the treatment of PDAC. Although these early clinical outcomes were mixed, they collectively demonstrated the biological activity and clinical feasibility of autophagy inhibition in PDAC, providing a foundation for exploration of additional rational combination strategies.

Despite early signs of biological activity, the clinical integration of first-generation lysosomotropic agents (e.g., CQ and HCQ) into oncologic practice has been hampered by significant biological and pharmacologic challenges. Post-hoc analysis of the MAPS trial (NCT01506973) revealed that HCQ treatment frequently yielded inconsistent and incomplete autophagy inhibition, even at maximum tolerated doses (400–600 mg BID) [122], suggesting HCQ lacks the systemic potency required for therapeutic efficacy in PDAC. Meanwhile, a similar retrospective analysis of the PACT trial (NCT01978184) found that a specific subset of patients deemed “biochemical responders” (defined by CA19-9 normalization) achieved significantly improved median survival compared to non-responders [119]. These observations together led to the hypothesis that autophagy inhibition is a viable treatment modality when it can be achieved, but the field currently lacks predictive biomarkers for identifying the specific patient populations most likely to derive clinical benefit.

Furthermore, recent evidence suggests that the suboptimal potency of HCQ may be partly driven by the unique stromal environment of PDAC tumors. As a weak base, HCQ must remain in an un-ionized state to permeate cell membranes; however, the acidic extracellular space of the PDAC tumor microenvironment (TME) triggers premature ionization of HCQ, effectively sequestering it in the stroma and preventing it from accumulating in the intracellular lysosomal compartment [123]. This effect is further compounded by extracellular matrix (ECM) proteins, which have been shown to serve as a physical barrier for ionized HCQ, further reducing its bioavailability to tumor cells [124]. These findings not only provide an important biological context for lysosomotropic treatment strategies but also suggest that autophagy inhibition may be more effective against lesions outside of the dense TME of PDAC, such as liver metastases or ascitic fluid.

#### Combination with MAPK inhibition

The therapeutic potential of autophagy inhibition is further underscored by its role as an adaptive bypass mechanism to therapeutic interventions in PDAC – most notably, the inhibition of MAPK signaling. Aberrant and constitutively active signaling of the mitogen-activated protein kinase (MAPK) is the primary oncologic driver of PDAC, and inhibition of this signaling cascade has been shown to induce autophagic flux as a compensatory survival mechanism [97]. Consequently, it was hypothesized that autophagy may represent a therapeutically exploitable emergent vulnerability following MAPK blockade in PDAC tumors. Indeed, two complementary studies demonstrated that MAPK inhibition – via KRAS suppression, direct ERK suppression [98], or pharmacologic MEK inhibition [97] – induces autophagy in pancreatic cancer cells. Dual pathway inhibition of MEK (trametinib) and autophagy (HCQ) produced synergistic anti-tumor effects in vivo and *in vitro*, and in one case, HCQ treatment led to a marked reduction in tumor burden and CA19-9 levels in a PDAC patient [97]. These landmark preclinical findings provided a compelling mechanistic rationale for this potentially synthetically lethal approach in human disease. While still in the early stages, data supporting the clinical implementation of this approach remains limited [125,126]. However, clinical trials are ongoing testing HCQ in combination with direct MEK (NCT03825289, NCT04132505) [127] and ERK (NCT04386057) inhibitors and will provide necessary insight into the biology and potential of this combination inhibition strategy. Parallel clinical studies evaluating HCQ with standard chemotherapy (mFOLFIRINOX; NCT05083780) and with immune checkpoint blockade (NCT04214418) are also underway, aiming to determine whether autophagy inhibition can sensitize PDAC to cytotoxic or immunomodulatory therapies.

#### Next-generation autophagy inhibitors

Building on the clinical foundation of HCQ, several next-generation autophagy inhibitors have been developed to target the pathway with increased potency and specificity. For example, ULK1/2, the nutrient-sensing kinase complex that initiates autophagy signaling, has emerged as a promising upstream target. DCC-3116, a selective ULK inhibitor, has shown potent anti-autophagy activity in preclinical models of PDAC [128] and is now being tested in combination with trametinib in a phase I/II trial (NCT04892017) for patients with RAS- or RAF-mutant advanced solid tumors [129]. The results of this trial will be of particular significance as they will provide key insights not only into the clinical efficacy of the approach, but also the biology of autophagy signaling, determining whether late-stage (via HCQ) or early-stage (via ULK) inhibition yields more robust anti-tumor responses.

Another promising compound, ezurpimtrostat (GNS561), is an orally available small molecule inhibitor of palmitoyl-protein thioesterase-1 (PPT-1), a lysosomal enzyme required for depalmitoylation and trafficking of lysosomal proteins [130]. PPT-1 is highly expressed in many cancer types [131], including PDAC, and its targeted inhibition results in disruption of lysosomal enzyme trafficking, cellular mislocalization of mTOR, and lysosomal de-acidification, thereby inhibiting late stages of autophagic degradation [132]. Although findings regarding efficacy are limited, in a phase I clinical trial, ezurpimtrostat demonstrated a favorable safety profile and induced a stabilization in CA19-9 in a subset of patients [133]. Collectively, these developments highlight the effort to exploit the dependency of PDAC tumors on autophagy. Nevertheless, early evidence suggests adaptive resistance and transient responses remain a barrier to durable clinical benefit [134], emphasizing the need for further characterization of compensatory signaling.

### Autophagy activators as a therapeutic strategy in pancreatic dysfunction

Although most translational efforts in pancreatic disease have focused on inhibiting autophagy in cancer, an opposing strategy – therapeutic activation of autophagy – has begun to gain traction in metabolic and inflammatory disorders, such as diabetes and pancreatitis [135–137]. Growing evidence suggests that impaired or dysregulated autophagic flux results in β-cell dysfunction, mitochondrial injury, and acinar cell stress, suggesting that restoration of autophagy could be cytoprotective in these diseases. However, currently available pharmacologic autophagy activators display diverse mechanisms of action and pleiotropic metabolic effects, underscoring the need for more specific and context-sensitive autophagy inducers. Current therapeutic strategies aimed at potentiating autophagy generally fall into two mechanistic classes: (1) mTOR-dependent activators, which act by suppressing mTORC1 signaling or enhancing AMPK activity to relieve inhibition of the ULK1 complex; and (2) mTOR-independent activators, which act directly on autophagy or lysosomal machinery, transcriptional regulators, or organelle-specific clearance pathways such as mitophagy (Figure 3).

#### mTOR-dependent activation of the ULK1/AMPK axis

Perhaps the most prominent therapeutic framework for autophagy activation is augmentation of the ULK1/AMPK/mTORc1 signaling axis, which integrates nutrient and energy status to regulate autophagosome initiation [138]. mTOR inhibitors such as rapamycin and everolimus relieve mTORc1-mediated suppression of the ULK1 complex, thereby enhancing autophagy activation. In preclinical models, mTOR inhibition increases autophagic flux and exhibits pancreas-preserving effects, including increased pancreatic insulin content, preservation of β-cell integrity [139,140], and amelioration of hypertriglyceridemia-related pancreatic injury [141] in mouse models of diabetes and pancreatitis, respectively. However, the translational potential of these findings remains complex; chronic mTOR suppression has been shown to induce hypertriglyceridemia and exacerbate acute pancreatitis [142], as well as impair insulin signaling, culminating in new-onset diabetes [143,144], suggesting mTOR inhibitors may be protective in short-term doses or tissue-specific contexts, but deleterious when used chronically or systemically.

Upstream of mTOR, AMPK promotes autophagy by phosphorylating ULK1 and leading to mTORc1 inhibition. As such, pharmacologic potentiation of AMPK has emerged as a strategy for inducing autophagy signaling. Indirect AMPK activators, such as metformin, have been shown to increase autophagic flux, preserve β-cell function, and improve insulin sensitivity in both preclinical models of diabetes [145]. Likewise, AICAR, an AMP mimetic, attenuates acinar cell necrosis and disease severity in experimental models of acute pancreatitis through AMPK-dependent autophagy activation [146]. Meanwhile, direct AMPK activators (e.g., MK-8722, PF-06409577) are currently in preclinical and early clinical development and have demonstrated promising activity in maintaining glucose homeostasis [147]. However, these compounds are associated with potentially severe toxicity profiles, including cardiac hypertrophy and dose-dependent glycogen accumulation [147], raising questions about their long-term safety.

#### mTOR-independent activators and peptide mimetics

Beyond canonical mTOR signaling, several compounds have also been identified that activate autophagy by acting directly on autophagic machinery or transcriptional regulators. For example, Tat-Beclin1 is a peptide mimetic of the autophagy protein Beclin-1 and has been shown to directly enhance autophagosome formation [148]. In pancreatic and hepatic injury models, Tat-Beclin1 treatment was shown to restore autophagic flux and reduce inflammatory damage, leading to improved cell viability [148]. Similarly, the disaccharide trehalose acts as an mTOR-independent activator of autophagy, enhancing the clearance of dysfunctional organelles and proteotoxic aggregates [149,150]. Although these agents have yet to make a clinical impact, their ability to enhance autophagic flux without the toxicities of direct mTOR inhibition makes them attractive candidates for pancreatic disorders characterized by defective autophagic degradation. Meanwhile, urolithin A, a naturally occurring and selective mitophagy activator, represents another promising avenue of autophagy modulation. In mice, urolithin A has been shown to preserve mitochondrial integrity and prolong lifespan [151], while human studies have demonstrated evidence of enhanced mitophagy associated with increased whole-body metabolic function [152]. Given the role of mitochondrial stress and injury in diabetes and pancreatitis, urolithin A and related mitophagy enhancers may warrant investigation in pancreatic models.

Transcriptional regulation of the autophagy-lysosome signaling network represents an additional axis of therapeutic intervention. Genetic knockout studies have established that expression of *TFEB*, the master regulator of lysosomal biogenesis [153], is necessary for the clearance of damaged organelles and protein aggregates and plays a critical protective role against the development of pancreatitis [54,154]. In preclinical pancreatitis models, transcriptional activation of *TFEB* via vagal stimulation has been found to enhance autolysosome fusion and degradation, prevent acinar cell injury, and reduce the severity of acute pancreatitis [155]. Finally, recent work found that dietary restriction in *C. elegans* correlates with more efficient autophagy and improved survival [156–158]. Therefore, strategies aimed at modulating intracellular nutrient-sensing pathways may also provide advantageous pro-autophagy effects in some contexts. Indeed, spermidine, a polyamine with TFEB-enhancing activity, has been shown to induce autophagy in response to fasting [159], providing yet another potential therapeutic avenue to rescue autophagy-mediated degradation.

## Conclusions

Autophagy is a dynamic process within the pancreas that is critical for the maintenance of normal pancreatic function. Modest activation of autophagy is protective from pathologic states such as pancreatitis and cancer; however, when autophagy is overactivated or severely impaired, it can lead to cellular destruction and pancreatic organ dysfunction. Although macroautophagy has been explored with numerous mouse models and genetic manipulations, the contribution of other forms of autophagy, such as chaperone-mediated autophagy, to pancreas physiology and pathophysiology remains relatively poorly understood. Additionally, exploring organelle-specific autophagy in a context-dependent manner in pancreatic physiology will be critical to a better understanding and potentially pharmacologic targeting of pancreatic pathophysiologic states. The pleotropic nature of autophagy will require further exacting studies in the future to better understand how to modulate autophagy for therapeutic benefit.

## Data Availability

Data sharing is not applicable to this article as no data were created or analyzed in this study.
